# A Peri-Ictal EEG-Based Biomarker for Sudden Unexpected Death in Epilepsy (SUDEP) Derived From Brain Network Analysis

**DOI:** 10.3389/fnetp.2022.866540

**Published:** 2022-04-26

**Authors:** Uilki Tufa, Adam Gravitis, Katherine Zukotynski, Yotin Chinvarun, Orrin Devinsky, Richard Wennberg, Peter L. Carlen, Berj L. Bardakjian

**Affiliations:** ^1^ Institute of Biomedical Engineering, University of Toronto, Toronto, ON, Canada; ^2^ Edward S. Rogers Sr. Department of Electrical and Computer Engineering, University of Toronto, Toronto, ON, Canada; ^3^ Department of Radiology and Medicine, McMaster University, Hamilton, ON, Canada; ^4^ Comprehensive Epilepsy Program and Neurology Unit, Phramongkutklao Hospital, Bangkok, Thailand; ^5^ Department of Neurology, New York University School of Medicine, New York, NY, United States; ^6^ Division of Neurology, Toronto Western Hospital, Toronto, ON, Canada; ^7^ Department of Physiology, University of Toronto, Toronto, ON, Canada

**Keywords:** epilepsy, SUDEP (sudden unexpected death in epilepsy), electroencephalography, brain dynamics, functional brain connectivity, brain networks, seizures

## Abstract

Sudden unexpected death in epilepsy (SUDEP) is the leading seizure-related cause of death in epilepsy patients. There are no validated biomarkers of SUDEP risk. Here, we explored peri-ictal differences in topological brain network properties from scalp EEG recordings of SUDEP victims. Functional connectivity networks were constructed and examined as directed graphs derived from undirected delta and high frequency oscillation (HFO) EEG coherence networks in eight SUDEP and 14 non-SUDEP epileptic patients. These networks were proxies for information flow at different spatiotemporal scales, where low frequency oscillations coordinate large-scale activity driving local HFOs. The clustering coefficient and global efficiency of the network were higher in the SUDEP group pre-ictally, ictally and post-ictally (*p* < 0.0001 to *p* < 0.001), with features characteristic of small-world networks. These results suggest that cross-frequency functional connectivity network topology may be a non-invasive biomarker of SUDEP risk.

## Introduction

Sudden unexpected death in epilepsy (SUDEP) is the leading cause of epilepsy-related mortality, however, the etiology remains poorly understood (Thurman et al., 2014; Devinsky et al., 2016; Sveinsson et al., 2017). Fear of SUDEP can decrease quality of life for patients and family members. There are no validated SUDEP risk biomarkers, which are needed to develop and assess interventions and prevention strategies for individuals and more broadly (Devinsky et al., 2016; Odom and Bateman 2018).

Several clinical factors correlate with SUDEP risk. A few of these, such as occurrence and frequency of generalized tonic-clonic and other types of seizures over the preceding year, duration of epilepsy, and use of multiple anti-seizure medications, among others (Novak et al., 2015), have been combined to form the SUDEP Risk Inventory (SUDEP-7), which provides a total score suggestive of overall SUDEP risk (Hesdorffer et al., 2011). Studies of SUDEP biomarkers have focused mainly on predicting SUDEP risk through findings correlating with SUDEP-7, other clinical risk factor algorithms or heart rate variability (Jha et al., 2021; Sivathamboo et al., 2021). Many of these biomarkers only have an indirect association with SUDEP (Odom and Bateman 2018; Ryvlin et al., 2019). Few studies prospectively assessed their predictive power (Novak et al., 2015; Ryvlin et al., 2019). SUDEP biomarkers with a more direct association may include peri-ictal cardiorespiratory dysfunction and prolonged post-ictal generalized electroencephalography (EEG) suppression (PGES), although findings are contradictory (Kang et al., 2017; Odom and Bateman 2018; Ryvlin et al., 2019). EEG based biomarkers, including prolonged electroclinical tonic phase and dynamics of seizure termination are correlated with PGES duration but have not been tested in a SUDEP patient cohort (Tao et al., 2013; Alexandre et al., 2015; Bauer et al., 2017; Grigorovsky et al., 2020). Delta-gamma cross-frequency interactions are a potential surrogate of PGES (Grigorovsky et al., 2020) and were found to persist during the peri-ictal period of a SUDEP patient. To date, studies exploring EEG SUDEP biomarkers have neglected measures targeting functionally aberrant connections in brain networks, which are characteristic of the epileptic brain.

Functional brain networks reflect the complex interactions in the brain and may distinguish pathology from normal functioning brain. These rhythms are important in information processing in the brain, with low frequencies being more spatially distributed and responsible in coordinating local high frequency activity. In this study, we aim to compare peri-ictal network differences in SUDEP patients using graph theory measures of directed functional connectivity. Specifically, we construct novel directed graphs combined from delta - HFO functional connectivity to capture the cross-frequency interactions between different brain regions. We use these directed graphs and their topologies to discern between SUDEP and non-SUDEP epileptic patients.

## Materials and Methods

### Data Acquisition

Scalp EEG recordings for 14 non-SUDEP (with 77 peri-ictal segments) and 8 SUDEP (with 25 peri-ictal segments) patients were provided through a consortium formed by the Toronto Western Hospital, the NYU Comprehensive Epilepsy Center, and the Phramongkutklao Royal Army Hospital (Table 1). Non-SUDEP patients had focal (temporal or extratemporal lobe) epilepsy, were resistant to anti-seizure medications and were undergoing presurgical evaluation. Ictal segments were marked by board-certified neurologists and electroencephalographers. The institutional review boards of the consortium approved the study protocol and all patients gave informed consent.

**TABLE 1:** Patient characteristics.

Patient	Classification	Age	Sex	Sampling Rate (Hz)	# of ictal Recordings	Length of ictal segments (s)
P1	non-SUDEP	28	M	500	2	75–128
P2	non-SUDEP	52	F	500	1	83
P3	non-SUDEP	56	-	512	1	135
P4	non-SUDEP	41	M	512	10	32–174
P5	non-SUDEP	19	M	512	4	113–138
P6	non-SUDEP	62	F	512	8	57–83
P7	non-SUDEP	42	F	512	2	19, 158
P8	non-SUDEP	40	F	512	7	30–129
P9	non-SUDEP	39	M	512	5	13–67
P10	non-SUDEP	28	M	512	8	54–109
P11	non-SUDEP	22	F	500	16	11–55
P12	non-SUDEP	30	F	500	4	76–182
P13	non-SUDEP	35	F	500	3	24–72
P14	non-SUDEP	31	F	500	6	5–27
P15	SUDEP	—	—	200	1	175
P16	SUDEP	—	—	256	3	81–92
P17	SUDEP	21	F	256	2	241, 744^a^
P18	SUDEP	26	F	512	1	63
P19	SUDEP	30	M	500	6	56–76
P20	SUDEP	43	F	512	6	45–283
P21	SUDEP	47	M	200	5	108–124
P22	SUDEP	30	M	256	1	38

a Part of the ictal duration during status epilepticus episode prior to medical intervention.

Patient data were originally filtered with a 0.1 Hz high pass filter during acquisition and were later pre-processed by removing power line interference using a finite impulse response (FIR) notch filter at 50 Hz or 60 Hz (data centre location dependent) and associated harmonics. Recordings used an acquisition reference at FCz, grounded at Fpz. Computations were performed on the Niagara supercomputer at the SciNet HPC Consortium. SciNet is funded by: the Canada Foundation for Innovation; the Government of Ontario; Ontario Research Fund - Research Excellence; and the University of Toronto (Loken et al., 2010; Ponce et al., 2019).

### Wavelet Phase Coherence

Wavelet phase coherence (WPC) was computed between pairs of scalp EEG electrodes. The phase of different frequency bands was extracted through the complex wavelet transform (Cotic et al., 2015). The Morlet complex wavelet transform was used with a mother wavelet of central frequency of 0.8125 Hz and bandwidth of 5 Hz, as previously used on EEG data (Grigorovsky et al., 2020). The relative phase difference was obtained using

$$\text{Δ}\phi \left(s,\;\tau \right)=\tan^{-1}\left(\frac{W_{1}^{*}\left(s,\tau \right)W_{2}\left(s,\tau \right)-W_{1}\left(s,\tau \right)W_{2}^{*}\left(s,\tau \right)}{W_{1}\left(s,\tau \right)W_{2}\left(s,\tau \right)-\;W_{1}^{*}\left(s,\tau \right)W_{2}^{*}\left(s,\tau \right)}\right)$$ (1)

where "W" is wavelet coefficient, $"W^{*}"$ is the complex conjugate, “s” is the scaling coefficient and "τ" is the time shift. The phase coherence between two electrodes is computed over a time window (N·Δt) expressed as an integer multiple N of the sampling period Δt . The phase coherence is defined as:

$$\rho \left(s,\;\tau \right)=\left|\langle e^{j\text{Δ}\phi \left(s,\tau \right)}\rangle \right|=\frac{1}{N+1}\overset{N/2}{\underset{k=-N/2}{\sum }}e^{j\text{Δ}\phi \left(s,\tau +k\text{Δ}t\right)}$$ (2)

WPC was applied to each wavelet central frequency in the delta (0.5–2 Hz) and HFO (80–120 Hz) ranges, in increments on a logarithmic base two scale. Wavelet frequency scales took the form of $2^{k}$ where "k" ranged from −2.0 to 1.0 for the delta range and 6.3 to 6.9 for the HFO range in 0.1 incremental steps. The WPC window size was proportional to 8 cycles for each frequency.

The undirected connectivity matrices were computed at 1 s intervals by assigning each edge corresponding to a pair of scalp electrodes to the WPC averaged over the frequency range (delta or HFO) and over 1 s temporal windows. The edges between electrodes are undirected, yielding to symmetrical connectivity matrices.

$$e_{ij}\left(t\right)=e_{ji}\left(t\right)=\langle \rho_{ij}\left(s,\tau \right)\rangle \;|\;\;e_{ij}\in E,\;\left(i,j\right)\in V^{2}$$ (3)

where " $e_{ij}\left(t\right)"$ and $"\rho_{ij}\left(s,\tau \right)"$ is the edge and WPC between node "i " and "j" , "E" and " V" is the set of all edges and the set of vertices respectively in the graph.

### Directed Low to High Frequency Connectivity

The undirected connectivity matrices were derived from delta–HFO WPC. The degree of each node was computed for both the delta and HFO WPC connectivity adjacency matrices to construct the directed delta-HFO network. Edges between pairs of nodes i and j were computed from the product of the delta degree of node i and HFO degree of node j . This measure represents information flow across the brain pertaining to the coexistence of simultaneous cohered delta and cohered HFO regions, which are two frequency ranges associated with seizure activity (Guirgis et al., 2015; Grigorovsky et al., 2020). The nature of this simultaneous coexistence may or may not be associated (Figure 2) with classical cross-frequency coupling (Tort et al., 2008; Canolty and Knight 2010; Stankovski et al., 2017). Additionally, this measure can identify phase-amplitude cross-frequency coupling (Supplementary Figure S1), where low frequency oscillations coordinate large scale activity driving local HFOs.

### Graph Theory Measures

Global coherence was computed as a measure for the undirected delta and HFO connectivity networks. First the eigenvalues of the connectivity matrix were computed and sorted. The global coherence is a ratio between the largest eigenvalue to the sum of all eigenvalues and has been previously used to analyze spatiotemporal EEG dynamics (Cimenser et al., 2011).

$$C_{Global}=\frac{\lambda_{\max\;}}{\sum^{\text{​}}\lambda }$$ (4)

The temporal mean of the global coherence was used to assess group differences.

Two network measures were computed: the clustering coefficient and the global efficiency. Together, these measures provide a description of the connection topology in the brain network.

The clustering coefficient for each node/electrode "C→i" in the directed graph is defined as the fraction of directed edges between adjacent nodes of node i over the maximum amount of directed edges

$${\vec{C}}_{i}=\frac{\frac{1}{2}\sum_{j,h\in V}\left(e_{ij}+e_{ji}\right)\left(e_{ih}+e_{hi}\right)\left(e_{jh}+e_{hj}\right)}{\left(k_{i}^{\mathrm{out}}+k_{i}^{in}\right)\left(k_{i}^{\mathrm{out}}+k_{i}^{in}-1\right)-2\sum_{j\in V}e_{ij}e_{ji}}$$ (5)

where $"k_{i}"$ is the degree of node i (Watts and Strogatz 1998; Fagiolo 2007; Liao et al., 2011). The clustering coefficient of the network is the mean clustering coefficient of all nodes.

$$C=\frac{1}{n}\underset{i\in V}{\sum }{\vec{C}}_{i}$$ (6)

The global efficiency of the network is a measure which quantifies how information flows throughout the network. Graphs with a high global efficiency have on average shorter paths connecting any two nodes within the network. The global efficiency was used instead of the characteristic path length as the shortest path length is not defined when a network contains two nodes that are not connected by any path. The global efficiency is the average efficiency, defined as the inverse of the shortest path between two nodes, over all electrode pairs (Latora and Marchiori 2001; Mitsis et al., 2020).

$$E=\frac{1}{n\left(n-1\right)}\sum_{i,j\in N,\;i\neq j}\frac{1}{d_{ij}}$$ (7)

where $"d_{ij}"$ is the shortest path between nodes i and j in the directed graph. Graph theory measures were computed using the Brain Connectivity Toolbox in Python (Rubinov and Sporns 2010). Graph visualization was created using the circular layout graph of the MNE-Python software package (Gramfort et al., 2013).

### Statistical Analysis

DABEST Python toolbox was used for two group comparisons of graph measures generating Gardner-Altman estimation plots for independent group mean differences (Ho et al., 2019). Bootstrapping was used to obtain distribution and confidence intervals for difference in groups. The Wilcoxon rank sum test was also used to test for significance between the two groups, as a separate test from the bootstrapping confidence intervals.

## Results

Abnormalities of brain networks have been implicated in different brain disorders including epilepsy (Liao et al., 2010). In this study, we explore network properties as a biomarker of SUDEP. 1) We constructed functional connectivity networks from delta and HFO WPC. 2) These networks were combined to create cross-frequency directed graphs as a proxy for information flow in different spatiotemporal scales of the brain. The directed graphs were validated using simulated data and seizure examples from a SUDEP and non-SUDEP epileptic patient. 3) We compared the topological differences in the directed networks between the two groups, yielding in a biomarker for SUDEP.

### Functional Connectivity Network

**FIGURE 1:**
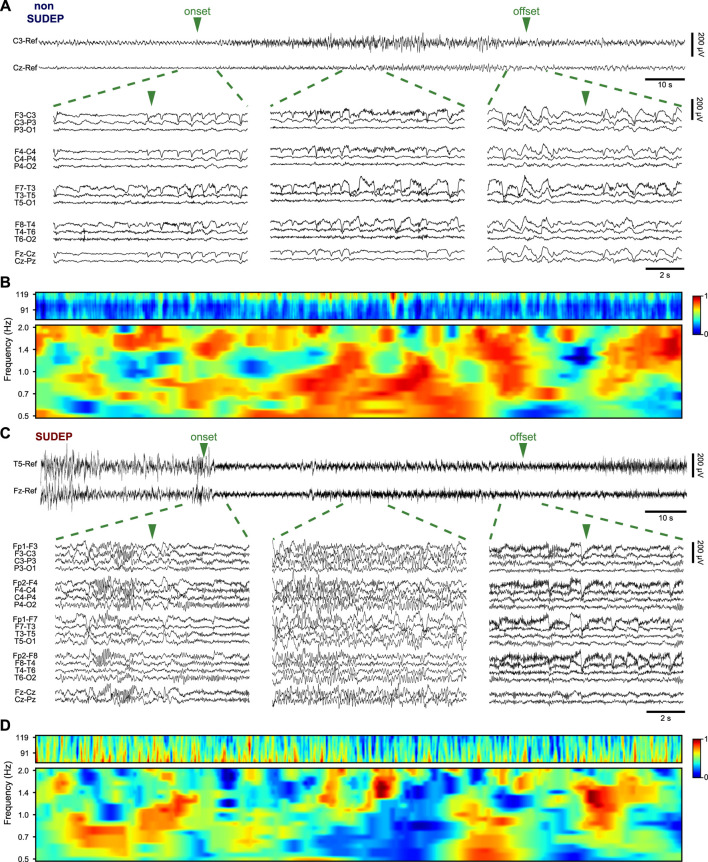
**(A)** Scalp EEG recording of a seizure from a non-SUDEP patient (P2) and **(B)** the corresponding delta (0.5–2 Hz) and HFO (80–120 Hz) wavelet phase coherence of electrodes C3 and Cz. **(C)** Scalp EEG recording of a seizure from SUDEP patient (P19) and **(D)** the corresponding wavelet phase coherence of electrodes T5 and Fz. For (A) and (C) the two electrodes are shown in referential montage while zoomed in regions of the seizure show all electrodes in a bipolar montage. Low-frequency filter 0.5 Hz; high-frequency filter 120 Hz.

FCN gives insight into disease induced changes in synaptic plasticity and efficiency of communication within neural networks in the brain (Bettus et al., 2008). WPC has previously been used as a measure for FCN in the brain, depicting coupling of different brain regions by way of specific brain rhythms (Cotic et al., 2015). Figure 1 shows scalp EEG traces of representative seizures in a non-SUDEP (P2) and SUDEP patient (P19), and examples of the corresponding WPC between two electrodes. The chosen electrodes showed the highest closeness centrality during seizure. Differences in the WPC distributions reaffirmed the choice of the two frequency ranges. The analysis was repeated for each pair of nodes and averaged over the frequency range and temporal windows to provide the connectivity strength between the two nodes.

### Validation of the Cross-Frequency Directed Graph Connecting Low and High Frequency Hubs

**FIGURE 2:**
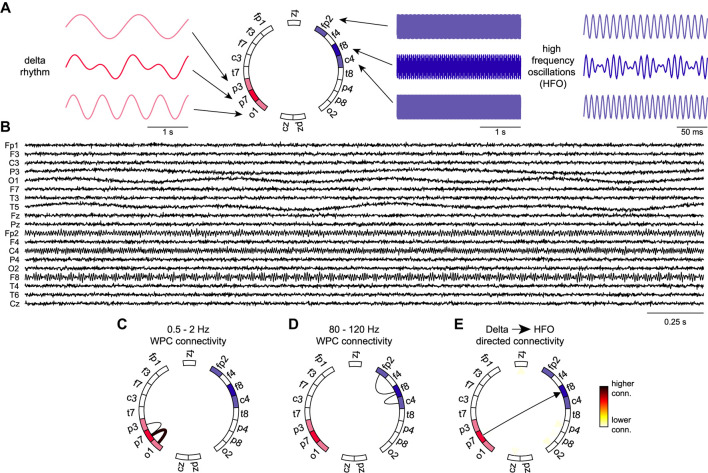
Validation of the directed graph measure using simulated EEG rhythms **(A)** Simulated delta rhythms with different frequencies are added to electrodes O1 and P3 with the combined rhythm added to P7. Simulated HFO rhythms with different frequencies are added to electrodes Fp2 and C4 with the combined rhythm added to F8. The design of the simulated network sets the P7 electrode as the strongest source of low frequency activity and the F8 electrode as the strongest source of high frequency activity. **(B)** Simulated EEG traces by adding the rhythms in (A) to the appropriate channels and adding Gaussian white noise to every trace. **(C–D)** Functional connectivity graphs with adjacency matrices computed by averaging the wavelet phase coherence between pairs of electrodes over the entire traces and over delta and HFO frequency ranges respectively. **(E)** Directed connectivity graph computed from connectivity graphs in C and D. Edges between pairs of electrodes *i* and *j* (nodes) are computed from the product of the delta degree of electrode *i* and HFO degree of electrode *j*. The directed graph accurately represents the connection between the low frequency hub to the high frequency hub and the direction of information flow.

**FIGURE 3:**
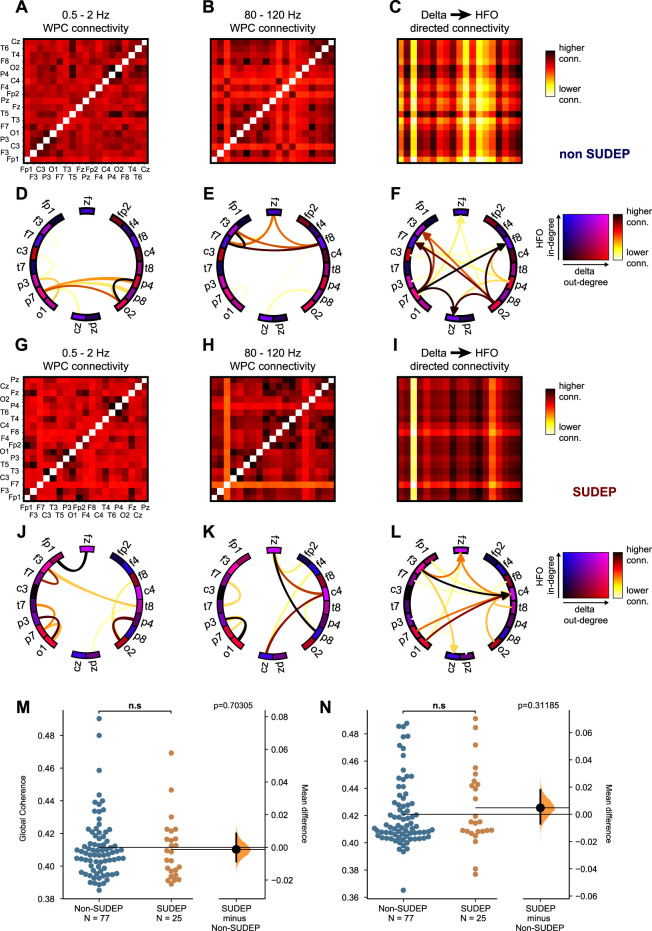
**(A–B)** Functional connectivity adjacency matrices computed by averaging the wavelet phase coherence between pairs of electrodes over the electrographic seizure and over delta and HFO frequency ranges respectively in non-SUDEP patient. **(C)** Directed connectivity adjacency matrix computed from connectivity matrices in A and B. Edges between pairs of electrodes *i* and *j* (nodes) are computed from the product of the delta degree of electrode *i* and HFO degree of electrode *j*. This measure represents information flow across the brain pertaining to delta-HFO coupling, where low frequency oscillations are important in coordination of large-scale activity driving more local high frequency oscillations. **(D–F)** Graph diagrams corresponding to connectivity matrices in A-C highlighting edges having the highest strength. Graph nodes are colored based on their ranking of delta and HFO degree. Similarly, **(G–I)** shows the functional connectivity matrices in a SUDEP patient, **(I)** the directed connectivity matrix, and **(J–L)** corresponding graph visualizations. Mean delta **(M)** and HFO **(N)** global coherence during the ictal period is unable to distinguish SUDEP from non-SUDEP (Wilcoxon rank sum test, *p* > 0.05).

Complex information flow involves multi-frequency large-scale organization in the brain (Buzsaki 2006; Jirsa and Müller 2013). We used directed networks to provide information about the coupling directionality between low and high frequency rhythms. The directed graph is constructed using the low frequency and high frequency with edge weights corresponding to the product of the low frequency graph degree of one electrode to the high frequency graph degree of another electrode. To validate our cross-frequency directed network, we simulated EEG rhythms and low/high frequency hubs. Starting with an empty set of EEG signals, we began populating specific channels with chosen delta and HFO rhythms. All channels contained Gaussian white noise. We aimed to create one low frequency hub and one high frequency hub and confirm that the directed connectivity network showed a directed edge between them. The low frequency hub was chosen to be electrode P7. This hub was designed to contain two different delta rhythms which then would each spread to another electrode (P3 and O1). The high frequency hub was chosen to be electrode F8. This hub was designed to contain two different HFO rhythms which then would each spread to a nearby electrode (Fp2 and C4). As expected, the low and high frequency networks shown in Figures 2C,D showed the desired connections. The directed connectivity network correctly identified the connection between the low and high frequency hubs as per our network design. Furthermore, the network analysis proved to be consistent when applied to recorded EEG data from a SUDEP (P19) and non-SUDEP patient (P1) (Figure 3). Taken together, these results validate and demonstrate how the directed graph captures cross-frequency interactions within the brain.

### Temporal Changes of the Delta-HFO Directed Network During Seizure

**FIGURE 4:**
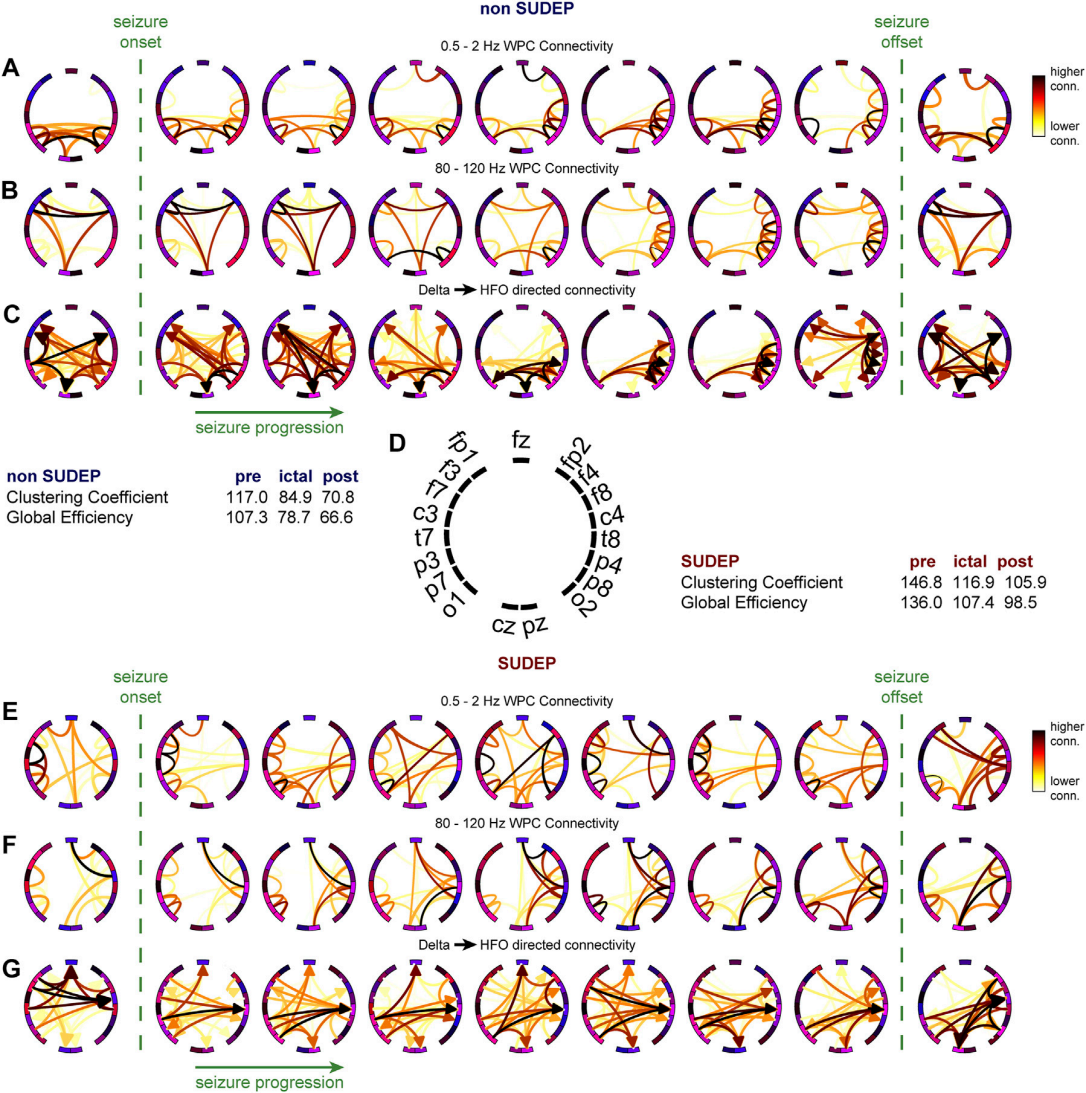
Dynamic changes in the ictal delta-HFO network not found in SUDEP patients. **(A,B)** Undirected functional connectivity computed from average wavelet phase coherence between each pair of electrodes using a 10 s sliding window throughout a seizure event in a non-SUDEP patient. **(C)** Directed graph computed using the delta and HFO graphs, connecting nodes with strong low-frequency degrees to nodes with strong high-frequency degrees. **(E,F)** Undirected functional connectivity computed from average wavelet phase coherence between each pair of electrodes using a 10 s sliding window throughout a seizure event in a SUDEP patient. **(G)** Directed graph computed using the delta and HFO graphs. Note the difference in seizure network dynamics of the directed graphs in the non-SUDEP patient in **(C)** compared to the SUDEP patient in **(G)**.

We constructed the delta-HFO directed network for consecutive 10-s duration time windows during peri-ictal regions in representative SUDEP (P19) and non-SUDEP (P1) patients (Figure 4). The graph visualizations indicated differences in network seizure dynamics in a non-SUDEP patient that were not observed in a SUDEP patient (Figure 4). The directed graphs (Figures 4D–G) further highlighted the seemingly unchanging network of the non-SUDEP patient during the ictus. Topological measurements of the directed graphs were used to compare the networks between the two patients. The pre-ictal, ictal, and post-ictal mean clustering coefficient and global efficiency were higher in the SUDEP than in the non-SUDEP patient (Figure 4D).

### Peri-Ictal Topological Network Changes as Biomarker for SUDEP

**FIGURE 5:**
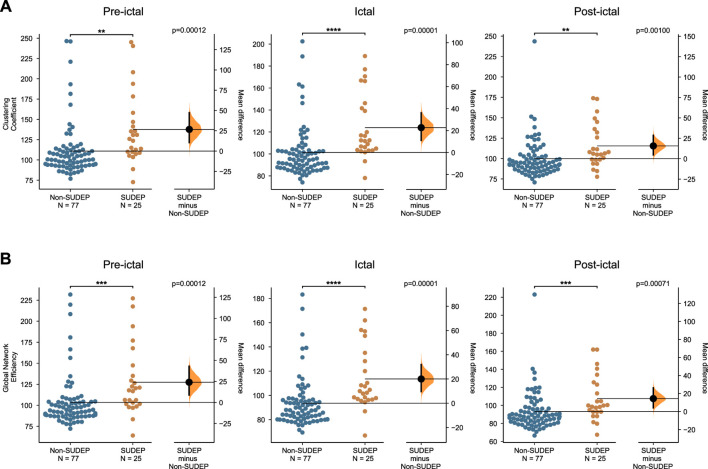
Group differences **(A)** in the mean clustering coefficient, and **(B)** in the mean global efficiency, between seizures of non-SUDEP and SUDEP groups pre-ictally, ictally, and post-ictally (Wilcoxon rank sum test, *p < 0.05, **p < 0.01, ***p < 0.001,****p < 0.0001).

The clustering coefficient and global efficiency measures were used to compare group differences between non-SUDEP and SUDEP epileptic patients (Figure 5). The clustering coefficient is an average measure of how node triples are connected within the network and specifies the tendency for nodes to cluster together. The clustering coefficient was significantly higher in the SUDEP group during the pre-ictal, ictal, and post-ictal segments (pre-ictal: *p* = 0.00100, ictal: *p* = 0.00001, post-ictal: *p* = 0.00100). The global efficiency, which indicates how efficiently information is transferred between nodes, was shown to be significantly higher in the SUDEP group pre-ictally, ictally, and post-ictally (pre-ictal: *p* = 0.00012, ictal: *p* = 0.00001, post-ictal: *p* = 0.00071). These results show that network topology is a potential biomarker in assessing SUDEP risk.

## Discussion

We have found topological differences in the peri-ictal delta-HFO directed networks of epileptic patients with SUDEP exhibiting significantly higher pre-ictal, ictal, and post-ictal clustering coefficient and global efficiency in the delta-HFO directed networks. These data suggest a higher connectivity and more efficient flow of information in seizure networks of SUDEP patients. Both high clustering coefficient and high global efficiency are features that resemble a small-world organized network as first described by Watts and Strogatz (Watts and Strogatz 1998). These networks are both locally and globally efficient, combining high clustering and short characteristic path length features (Latora and Marchiori 2001). The observed network changes suggest that cross-frequency network topology is a possible SUDEP biomarker.

The delta-HFO directed networks captured the complexity of the seizure networks and differences between SUDEP and non-SUDEP groups. The importance of these rhythms is consistent with previous studies localizing seizure networks (Cotic et al., 2015). A recent study from our group described delta-gamma cross-frequency coupling as a biomarker of PGES (Grigorovsky et al., 2020).

Proposed mechanisms of SUDEP involve ictal-related cardio-respiratory dysfunction, which may be caused by epileptiform activity spreading to the brainstem. A crucial element of SUDEP is brainstem dysfunction, for which PGES might be a biomarker (Lhatoo et al., 2010; Devinsky et al., 2016). The MORTEMUS study, which examined SUDEP cases that occurred in epilepsy monitoring units, found the cause of death to be due to postictal respiratory impairment and bradycardia (Ryvlin et al., 2013). The cross-frequency network differences that were observed in our study may suggest that the network is more efficient in the spread of seizure activity, reaching central autonomic structures more easily. This may increase the likelihood of ictal associated bradycardia and asystole. Spread to the brainstem may also affect respiration centers, inducing hypoxia and hypercapnia. This is consistent with findings where electrical stimulation of the amygdala induced respiratory arrest (So 2008).

Further research needs to be done using intracranial EEG in both patient groups to have a deeper understanding of how these topological changes relate to seizure spread and brainstem dysfunction. A limitation of this study is the low number of patients in the SUDEP group. More patients need to be added to the sample size to examine the predictive power of this biomarker. Although the network dynamics throughout representative seizures seemed to differ in SUDEP patients, further exploration needs to be done in comparing group differences. Regarding patient selection criteria, EEG recordings in this study were obtained from patients monitored in the EMU. While most patients were weaned off anti-seizure medications in order to provoke seizures, changes in their medication regimen were not annotated in the EEG recordings. This should be taken into consideration for future studies. Also, all non SUDEP patients were medically refractory and this may limit the generalizability of this biomarker to the broader epilepsy population. In conclusion, there is an unmet need for non-invasive biomarkers to identify those patients at high risk for developing seizure-associated SUDEP. Our study describes such a biomarker for SUDEP using scalp EEG signals to construct functional connectivity networks of the brain.

## Data Availability

The anonymized datasets used in this study are available upon request. They are not publicly available due to institutional restrictions associated with original data acquisition protocols.
