# Supplementary material for: A Peri-Ictal EEG-Based Biomarker for Sudden Unexpected Death in Epilepsy (SUDEP) Derived From Brain Network Analysis
Source: Front Netw Physiol. 2022 Apr 26;2:866540. doi: 10.3389/fnetp.2022.866540 (PMC10013055; doi:10.3389/fnetp.2022.866540)
Supplement: Supplementary file 1 [file DataSheet1.docx]

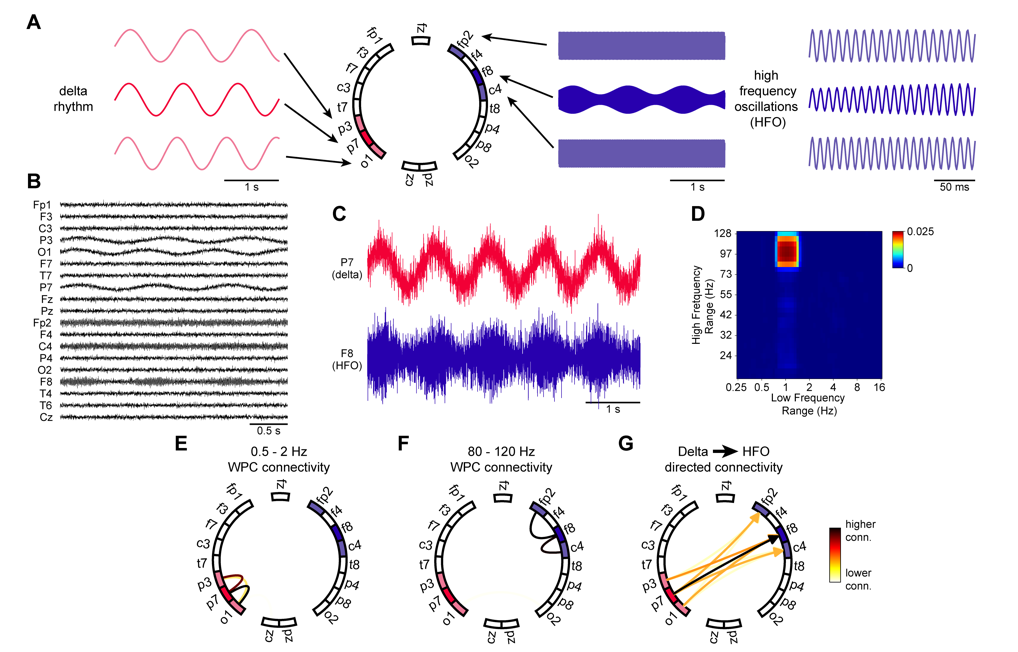


**Supplementary Figure 1**. Validation of the directed graph measure using simulated phase-amplitude cross-frequency coupled EEG rhythms (A) Simulated delta rhythms with different frequencies are added to electrodes O1, P3 and P7. Simulated HFO rhythms with different frequencies are added to electrodes Fp2, C4, and F8, with the amplitude of the HFO coupled to the phase of the delta rhythm of P7 electrode. (B) Simulated EEG traces by adding the rhythms in (A) to the appropriate channels and adding Gaussian white noise to every trace. (C) P7 and F8 channels showing delta-HFO phase-amplitude cross-frequency coupling where the phase of the delta rhythm in P7 modulates the amplitude of the HFO rhythm in F8. (D) Comodulogram showing phase-amplitude coupling strength between P7 and F8. (E-F) Functional connectivity graphs with adjacency matrices computed by averaging the wavelet phase coherence between pairs of electrodes over the entire traces and over delta and HFO frequency ranges respectively. (G) Directed connectivity graph computed from connectivity graphs in C and D. Edges between pairs of electrodes *i* and *j* (nodes) are computed from the product of the delta degree of electrode *i* and HFO degree of electrode *j*. The directed graph accurately represents the coupling between the low frequency rhythm in P7 to the high frequency rhythm in F8 and the direction of information flow.


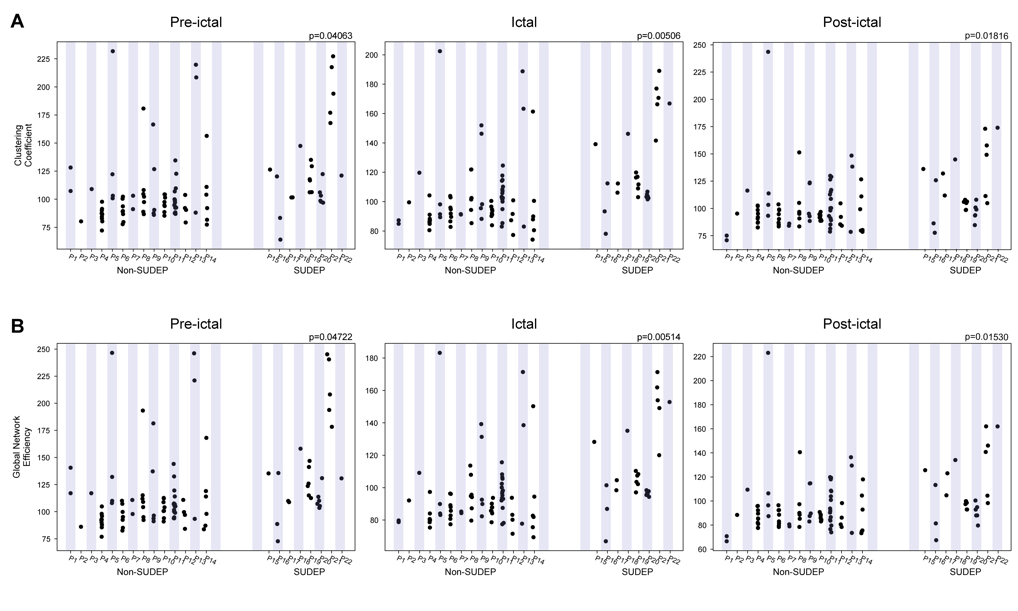
 **Supplementary Figure 2**. (A) Peri-ictal clustering coefficient and (B) global efficiency measures for individual patients. The median of the measures were used for individual patients to show statistical power in distinguishing SUDEP from non SUDEP groups (Wilcoxon rank sum test)
